# Evaluation of *Kynu*, *Defb2*, *Camp*, and *Penk* Expression Levels as Psoriasis Marker in the Imiquimod-Induced Psoriasis Model

**DOI:** 10.1155/2024/5821996

**Published:** 2024-07-16

**Authors:** Zahra Emami, Saeideh Sadat Shobeiri, Razia Khorrami, Navideh Haghnavaz, Mohammad Ali Rezaee, Malihe Moghadam, Safoora Pordel, Mojtaba Sankian

**Affiliations:** ^1^ Immunology Research Center Faculty of Medicine Mashhad University of Medical Sciences, Mashhad, Iran; ^2^ Cellular and Molecular Research Center Sabzevar University of Medical Sciences, Sabzevar, Iran; ^3^ Department of Medical Laboratory Sciences Faculty of Paramedical Kurdistan University of Medical Sciences, Sanandaj, Iran

## Abstract

**Background:**

Psoriasis is a noncontagious auto-inflammatory chronic skin disease. So far, some of the inflammatory genes were upregulated in mouse model of psoriasis. This study examined changes in skin mRNA expression of L-kynureninase (*Kynu*), cathelicidin antimicrobial peptide (*Camp*), beta-defensin 2 (*Defb2*), and proenkephalin (*Penk*) in a mouse model of imiquimod-induced psoriasis.

**Materials and Methods:**

Tree groups of C57BL/6 female mice were allocated. The imiquimod (IMQ) cream was administered to the mice dorsal skin of the two groups to induce psoriatic inflammation. In the treatment group, IMQ was administered 10 min after hydrogel-containing M7 anti-IL-17A aptamer treatment. Vaseline (Vas) was administered to the negative control group. The psoriatic skin lesions were evaluated based on the psoriasis area severity index (PASI) score, histopathology, and mRNA expression levels of *Kynu*, *Camp*, *Defb2*, and *Penk* using real-time PCR. In order to assess the systemic response, the spleen and lymph node indexes were also evaluated.

**Results:**

The PASI and epidermal thickness scores were 6.01 and 1.96, respectively, in the IMQ group, and they significantly decreased after aptamer administration to 1.15 and 0.90, respectively (*P* < 0.05). Spleen and lymph node indexes showed an increase in the IMQ group, followed by a slight decrease after aptamer treatment (*P* > 0.05). Additionally, the mRNA expression levels of *Kynu*, *Defb2*, *Camp*, and *Penk* genes in the IMQ-treated region showed a significant 2.70, 4.56, 3.29, and 2.61-fold increase relative to the Vas mice, respectively (*P* < 0.05). The aptamer-treated region exhibited a significant decrease in these gene expression levels (*P* < 0.05). A positive correlation was found between *Kynu*, *Penk*, and *Camp* expression levels and erythema, as well as *Camp* expression with PASI, scaling, and thickness (*P* < 0.05).

**Conclusion:**

According to our results, it seems that *Kynu*, *Camp*, and *Penk* can be considered appropriate markers for the evaluation of psoriasis in IMQ-induced psoriasis. Also, the anti-IL-17 aptamer downregulated these important genes in this mouse model.

## 1. Introduction

Psoriasis disease is a noncontagious chronic inflammatory skin condition that may be triggered by a trauma, infection, or drugs [1]. The initiation and persistence of psoriatic inflammation contribute to the dysfunction of both adaptive and innate immune reactions in the skin. Therefore, psoriasis is an autoimmune and autoinflammatory disease [2, 3]. The prevalence of psoriasis is about 2%–4% of the worldwide population [4]. Psoriasis includes a variety of types, with psoriasis vulgaris that is known by clinical manifestations, such as erythema, skin thickening, and scaling, being the most common type [5, 6, 7].

**Figure 1:**
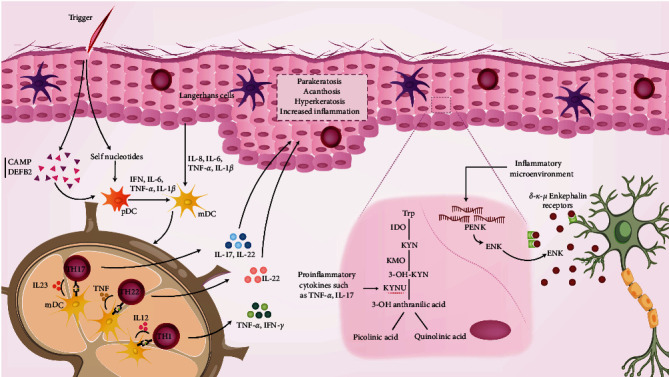
The association between psoriasis and KYNU, CAMP, DEFB2, PENK. KYNU, kynureninase, CAMP, cathelicidin antimicrobial peptide, DEFB2: beta-defensin 2, PENK, proenkephalin, mDc, myeloid dendritic cell, TNF-*α*, tumor necrosis factor-alpha, IL, Interleukin, IFN, interferon, pDC, plasmacytoid dendritic cell, KYN, kynurenine, Trp, tryptophan, KMO, kynurenine 3-monooxygenase, ENK, enkephalin, TH, T-helper cell, IDO, indoleamine 2,3-dioxygenase.

Various factors stimulated keratinocytes in the inflammatory milieu of psoriasis disease. Stimulated keratinocytes produce various inflammatory mediators that are important in the development and persistence of psoriasis vulgaris lesions; these mediators include cytokines, chemokines, antimicrobial peptides (AMPs), endogenous opioid-like peptides, enzymes, etc. [8, 9, 10, 11]. The association between psoriasis and some of these mediators, such as L-kynureninase (KYNU), cathelicidin antimicrobial peptide (CAMP), beta-defensin 2 (DEFB2), and proenkephalin (PENK) is illustrated in Figure 1.

AMPs are first-line defense with a crucial role in the skin by killing pathogenic microorganisms [12, 13]. CAMP and DEFB2 are two types of AMPs secreted by keratinocytes in psoriasis [14]. LL37, which interacts with deoxyribonucleic acid (DNA) or ribonucleic acid (RNA), releases from apoptotic or necrotic cells and stimulates dendritic cells (DCs) to secrete inflammatory cytokines [15]. On the other hand, DEFB2 has been shown to induce proliferative reactions as well as promote the secretion of inflammatory factors in keratinocytes [16]. Therefore, it seems that the expression levels of both *CAMP* and *DEFB2* elevate in lesional skin compared to healthy skin [14].

Enkephalin (ENK) is involved in nociception and pruritus [17, 18], which is identified as a contributing factor to inflammation, which belongs to the family of endogenous opioid-like peptides. PENK is a protein precursor of ENKs [19, 20], which has been found to facilitate an interaction between the neuroendocrine and immune systems [21]. The PENK receptors are expressed on keratinocytes and regulate keratinocyte activities [22]. They have direct antimicrobial activities and regulate cell proliferation and differentiation [23]. The increase of PENK in psoriatic skin keratinocytes has been reported by studies [21, 23, 24].

Another factor involved in inflammation is KYNU, which is upregulated in psoriatic lesions [25]. The pathway of tryptophan metabolism includes various enzymes, one of them being KYNU [26]. Tryptophan metabolism, when mediated by the KYNU, causes inflammation. Kynurenine (KYN) is a metabolite of tryptophan that reduces the expression of some inflammatory genes in keratinocytes [25]. It has been observed that the upregulation of KYNU leads to the destruction of KYN and induces excessive inflammation in psoriasis [27].

Skin problems caused by psoriasis can be extremely burdensome sometimes. So far, there are various treatments known for psoriasis, and one type of topical agents for modulating of psoriasis symptoms is aptamers [28]. Aptamers are RNA and DNA (single-stranded form) molecules with 3D unique structures that specifically target biological molecules with a high affinity and specificity [29]. One of the important cytokines with a main role in the development of psoriasis is IL-17A [30]. M7 anti-IL-17A aptamer has been developed against IL-17A, which inhibits its cytokine effects and leads to a reduction in disease symptoms. So, it can be considered as a therapeutic agent that could prevent to engage of IL-17 with IL-17 receptor [31]. One of the psoriasis animal models is the imiquimod (IMQ)-induced psoriasis mouse model, which induces psoriasis using IMQ cream [32]. IMQ is an agonist of toll-like receptors (TLR) 7/8-ligand and induced lesions like psoriasis through this pathway [33].

It is very important to determine an appropriate marker to assess the induction of psoriasis caused by IMQ that can assess an adequate response after treatment with therapeutic agents. This study aimed to evaluate the psoriasis-correlated gene expression levels of *Kynu*, *Defb2*, *Camp*, and *Penk* in the IMQ-induced psoriasis mice skin and also in mice model treated with M7 anti-IL-17A aptamer for the purpose of determining a proper diagnostic marker for psoriasis and evaluating treatment response.

## 2. Materials and Methods

Twelve female C57BL/6 mice with a weight range of 18.3–26 g, aged 7–8 weeks, were obtained from the Pasture Institute (Tehran, Iran) and maintained under standard situations at Bu-Ali Research Institute (Mashhad, Iran). Ethical Committee of Mashhad University of Medical Sciences (IR.MUMS.ACE.1401.096) approved the animal experiment procedure.

### 2.1. Hydrogel Preparation

In order to prepare the gel, 40 ml water was poured into a container and stirred with a stirrer, followed by slowly adding 0.4 g of Carbopol (gel-forming carbomer powder) to the water. Then, 400 *µ*l of propylene glycol and 480 *µ*l of triethanolamine were added dropwise. After preparation, the resulting gel was collected with a spoon and poured into a tube.

### 2.2. Animals' Procedure and Grouping

All of the animals were divided into the following three groups randomly, with four mice in each group. About 2 cm^2^ of the mice dorsal skin was depilated using an electric shaver. In order to induce a psoriasis-like model, two experimental groups were received 40 mg (daily dose) of IMQ 5% cream (Aldara, UK). One group administered 40 mg/day of Vaseline (Vas) as the negative control group. This procedure was performed on the mice's dorsal skin topically, over a period of 5 consecutive days. The psoriasis area severity index (PASI) score and weight of the animals were recorded daily.

One group of IMQ-administered mice received the discovered aptamer targeting IL-17A named M7 (mebep, China) as the treatment group. The aptamer utilized in this study was identified in our previous research with the sequence 5′-CGACTAACTGTTTTCTTTTGTTTTTAGTCG-3′ (30-nt) [31].

**Figure 2:**
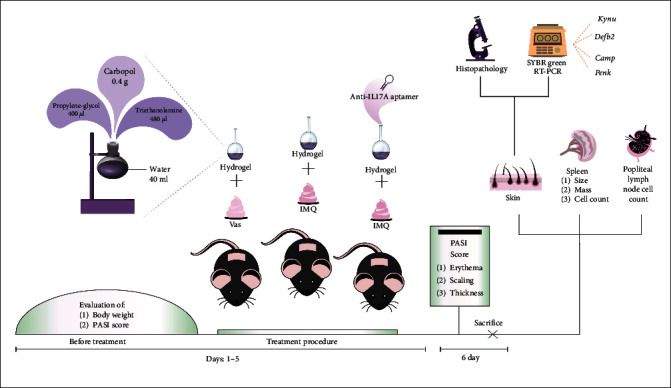
The procedure of the experiment. Vas, vaseline, IMQ, imiquimod, RT-PCR, real-time polymerase chain reaction, PASI, psoriasis area severity index, *Kynu*, kynureninase, *Camp*, cathelicidin antimicrobial peptide, *Defb2*, beta-defensin 2, *Penk*, proenkephalin.

All groups received 50 *µ*l of carbomer-based hydrogel. About 4 *µ*l of M7 anti-IL-17A aptamer (10 pmol) was topically applied along with the aforementioned hydrogel on the dorsal skin of the M7 anti-IL-17A aptamer group. IMQ or Vas is administered topically, 10 min after hydrogel application, once a day. Animals were sacrificed on the 6th day of the procedure, and their dorsal skin was isolated in order to histopathological and messenger RNA (mRNA) levels analyzing. Additionally, the mice popliteal lymph node and spleen were isolated for supplementary analysis [34]. The experiment procedure is illustrated in Figure 2.

### 2.3. PASI Score

In our study, the mice dorsal skin lesions were evaluated with the PASI score, which represent the prominent scoring in clinical symptoms measurement of psoriasis [35].

The PASI score, as an indicative of an inflammatory condition in psoriatic lesions, was evaluated in all mice before the treatments daily by two individuals who were blinded to the experimental groups. A numerical score between 0 and 4 (0—none, 1—light, 2—mild, 3—severe, and 4—very severe) was assigned for erythema, scaling, and thickness in mice's dorsal skin. The PASI cumulative score was presented as 0–12, determining the severity of psoriasis-related inflammation.

### 2.4. Popliteal Lymph Node

We labeled the popliteal lymph node using an injection of Evans blue (1%) dye into the mouse left footpad. Following the sacrifice of animals, the lymph node was isolated, and the cells were collected in PBS with physiologic pH (7.4). Subsequently, a hemocytometer chamber was utilized to count the cells/1 ml PBS.

### 2.5. Spleen Size, Mass, and Total Cell Count

Following the sacrifice of the animals, the spleens of all mice were isolated and subsequently evaluated for their mass, size (length), and total cell count. The mass of the spleen (in milligrams), size (in millimeters), and spleen cell count were measured using a digital scale (A&D in Japan), a ruler, and a hemocytometer counting-chamber, respectively. In order to assess the spleen total cell count, RBCs were lysed by lysis solution (NH_4_Cl: 0.01 M, NaHCO_3_ : 0.01 M, EDTA: 0.04 mM) and followed by washes with PBS, then the collected cells were adjusted up to a volume of 1 ml with PBS. The values were normalized according to the average of mice's last-day weight.

### 2.6. SYBR-Green Reverse Transcription Polymerase Chain Reaction

About 10 mg of mice dorsal skin tissue was separated. Following the kit manufacture's instruction (Parstous Co., Iran), the cellular RNA was extracted, and then, the complementary DNA (cDNA) synthesis was accomplished utilizing reverse transcriptase enzyme and oligo-dT primers (Parstous Co., Iran). Specific primers (SinaClon, Iran) were designed for *Kynu*, *Camp*, *Defb2*, and *Penk* genes (Table 1). The evaluation of *Kynu*, *Camp*, *Defb2*, and *Penk* genes mRNA levels was performed according to the guidelines provided by the SYBR-Green Master Mix kit (Parstous Co., Iran). Hypoxanthine phosphoribosyltransferase (Hprt) primers, which were designed by Shobeiri et al. [36] utilized in this study as a housekeeping gene. The real-time PCR procedure was performed in duplicate by a real-time PCR (Four E's Scientific, Guangzhou, China) detection system. In the relative gene expression results, 2^−*∆*Ct^ was calculated for each mouse, and then the 2^−*∆*Ct^ mean results of each group were divided by the Vas group 2^−*∆*Ct^ results mean and reported as fold change.

### 2.7. Histopathology

In the context of histopathological analysis, a part of the mice dorsal lesional skin was separated and fixed. The process of fixation involved the use of 10% formaldehyde, and then the sample was embedded within a matrix of paraffin blocks, cut, subsequently placed upon slides, and stained with hematoxylin and eosin (H&E) stain. In order to assess the mice skin epidermal thickness, the tissue sections were analyzed utilizing an optical microscope (BEL Photonics, Italy). In this study, epidermis length and microscopic region diameter were measured by ImageJ software version 1.44 (NIH, Bethesda, MD, USA), and the epidermal thickness score was calculated.

### 2.8. Statistical Analysis

The Graph-Pad Prism software (Version 8.4.2, California, USA) was employed for the analysis of data. Weight, spleen, and lymph node data were presented in the form of mean ± standard error (SEM). The data of spleen normalized with the mouse weight. Statistical analysis, including Mann–Whitney *U* and two-way ANOVA, was used to assess group differences. Spearman's correlation coefficient analysis was conducted to evaluate the correlation between our research inflammatory genes expression levels and erythema, scaling, thickness, and PASI. The *P*-value < 0.05 was considered significant.

## 3. Results

### 3.1. Evaluation of Weight Changes in Mice Groups

**Figure 3:**
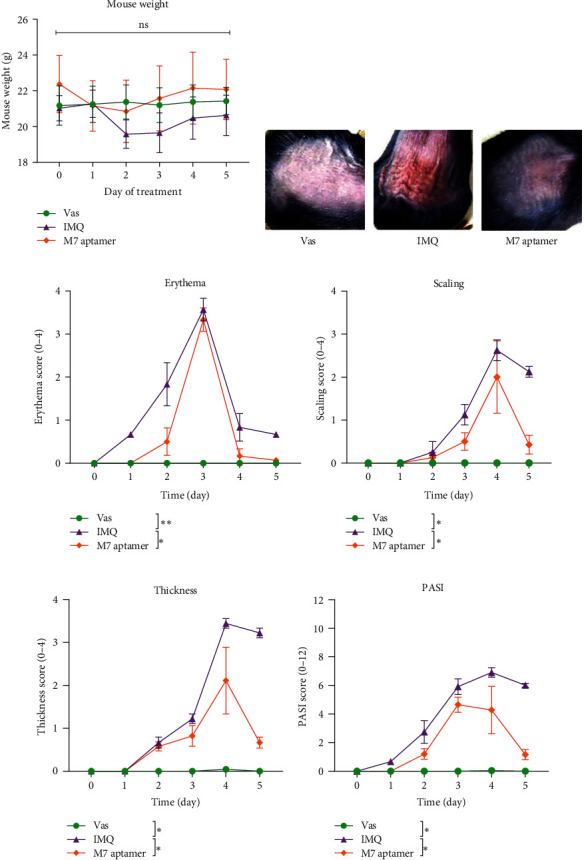
The evaluation of mice weight and PASI score. The measurements of mice weight on 5 consecutive days (a). Evaluation of dorsal skin manifestations following administered with Vas, IMQ, and M7 anti-IL-17A aptamer (b) indicated a significant increase in the IMQ group versus the Vas group, while a significant decrease was recorded in the treatments group versus the IMQ group with regards to the factors of erythema (c), scaling (d), thickness (e), and PASI score (f). All data of aforementioned parameters are shown as mean ± SEM. Vas, Vaseline (negative control), IMQ, Imiquimod, PASI, psoriasis area and severity index,  ^*∗*^*P*-value < 0.05,  ^*∗∗*^*P*-value < 0.01, ns, nonsignificant.

The evaluation of weight mean ± SEM by two-way ANOVA analysis in mice revealed no statistically significant alterations in the IMQ-treated mice in comparison to the Vas and the M7 anti-IL-17A aptamer-treated groups (Figure 3(a)).

### 3.2. Evaluation of PASI Score of Mice Groups

The mean ± SEM of erythema (Figure 3(c)), scaling (Figure 3(d)), thickness (Figure 3(e)), and PASI score (Figures 3(b) and 3(f)) in mice dorsal skin were assessed every day. The comparison of the aforementioned parameters 5th-day values average indicated a significant increase in the IMQ group versus the Vas group. The M7 anti-IL-17A aptamer-treated group findings demonstrated a significant decrease in erythema, scaling, thickening, and PASI score versus the IMQ group (*P*  < 0.05).

### 3.3. Evaluation of Spleen Size, Mass, and Total Cell Count in Mice Group

**Figure 4:**
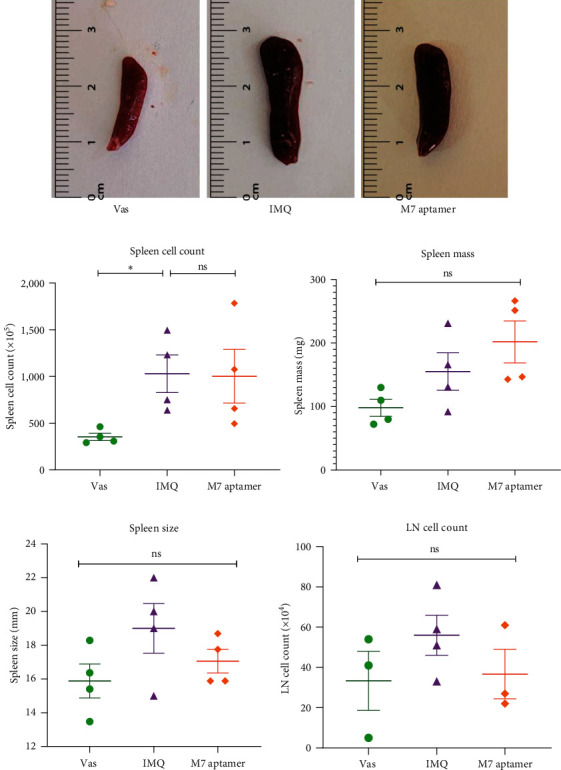
The evaluation of mice spleens and lymph nodes. Mice spleens were isolated from the Vas, IMQ, and M7 anti-IL-17A aptamer groups (a). Evaluation of mice following administration of Vas, IMQ cream, and M7 anti-IL-17A aptamer, showed the spleen cell count significantly increased in the IMQ group versus the Vas group (b). Evaluation of spleen mass (c), spleen size (d), and LN cell count (e) indicated an increase in the IMQ group compared to the Vas group. In contrast, the aptamer-treated mice showed a decrease in these parameters except spleen mass. Data are displayed as mean ± SEM. Vas, vaseline (negative control), IMQ, imiquimod, LN, lymph node,  ^*∗*^*P*-value < 0.05, ns, nonsignificant.

Evaluation of the spleen total cell count (Figure 4(b)) indicated a significant increase (*P*=0.021,) in the IMQ group ((1,030 ± 201.52) × 10^5^ cell/ml) compared to the Vas group ((354.25 ± 38.25) × 10^5^ cell/ml). Additionally, the statistical analysis of data revealed that the value for both spleen mass (mg, Figure 4(c)) and spleen size (mm, Figures 4(a) and 4(d)) showed a nonsignificant difference in the IMQ group (mass: 155 ± 29.49, size: 19 ± 1.47) as compared to the Vas group (mass: 97.95 ± 13.39, size: 15.88 ± 1.001). The results of our research indicate a nonsignificant difference in spleen size (17.05 ± 0.70), cell count ((1,003.45 ± 287.69) × 10^5^ cell/ml), and mass (201.81 ± 33.05) in the M7 anti-IL-17A aptamer group versus the IMQ group (*P*  > 0.05).

### 3.4. Evaluation of Popliteal Lymph Node in Mice Group

However, the analysis of popliteal lymph nodes (Figure 4(e)) revealed an increase in the count of lymph node cells in the IMQ group ((56 ± 9.94) × 10^4^ cell/ml) as compared to the Vas ((33.33 ± 14.65) × 10^4^ cell/ml) group and also, the results indicated a reduction in the M7 anti-IL-17A aptamer group ((36.66 ± 12.25) × 10^4^ cell/ml) in comparison with the IMQ group. These all results were nonsignificant (*P*  > 0.05).

### 3.5. Evaluation of Histopathology

**Figure 5:**
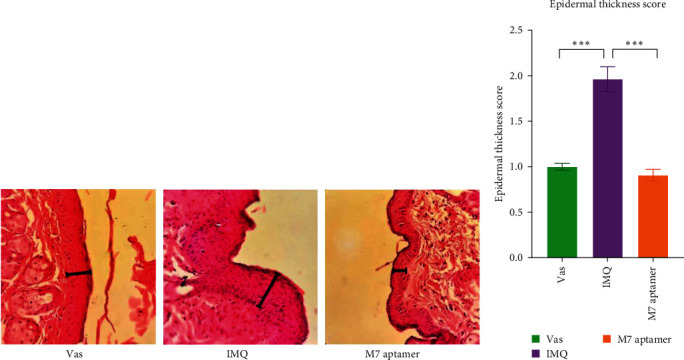
Histopathological analysis (H&E) of tissue sections on the mice dorsal skin (a). Statistical results of the epidermal thickness in the dorsal mice skin showed a significant elevation in the IMQ group and a significant reduction in the M7 anti-IL-17A aptamer (b). The value was displayed based on the epidermal length, and the microscopic region diameter was measured by ImageJ software. Data are displayed as relative to the Vas group. Vas, vaseline (negative control), IMQ, imiquimod,  ^*∗∗∗*^*P*-value < 0.001.

The present study utilized histopathological observations (Figure 5(a)) to confirm the results of the PASI score based on observations of dorsal skin lesions in mice. Statistical results (Figure 5(b)) revealed a significant increase in the epidermal keratinocyte proliferation of the IMQ group versus the Vas group (*P*  < 0.001). The experimental group that received the M7 anti-IL-17A aptamer demonstrated a significant decrease in the epidermal layer thickness as compared to the IMQ group (*P*  < 0.001).

### 3.6. Evaluation of the *Kynu*, *Defb2*, *Camp*, *Penk* mRNA Expression Levels

Evaluation of the mRNA levels of *Kynu*, *Camp*, *Defb2*, and *Penk* was carry out by SYBR Green RT-PCR. The Fold changes were computed for all groups relative to the Vas group.

**Figure 6:**
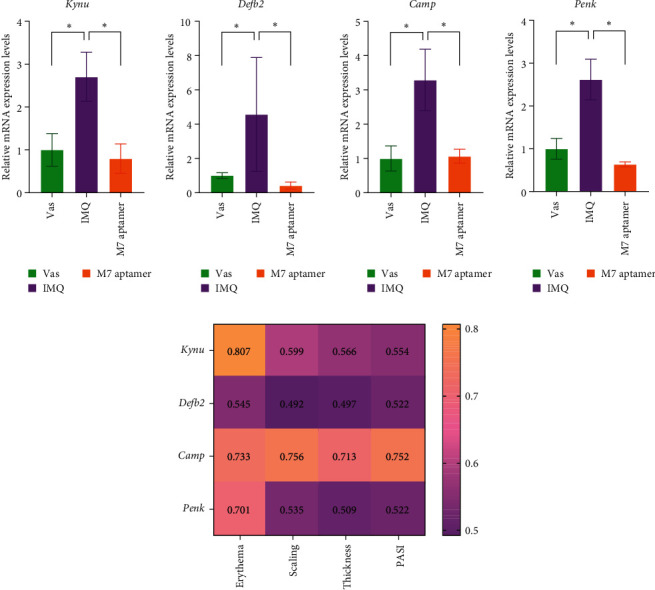
Relative mRNA expression levels of *Kynu* (a), *Defb2* (b), *Camp* (c), and *Penk* (d) analysis. Results indicated a significant increase in the IMQ group versus the Vas group and a significant decrease in the M7 anti-IL-17A aptamer compared to the IMQ group. The mRNA expression levels of the animal's dorsal skin were evaluated by SYBR green real-time PCR. Data are expressed as the fold change ± SEM. RT-PCR procedures were carried out in duplicate and repeated 3 times. Spearman's correlation between inflammatory genes and PASI, erythema, scaling, and thickness of the IMQ group was conducted, “*r*” presented in the heat map (e). Vas, vaseline (negative control), IMQ, imiquimod, *Kynu*, kynureninase, *Defb2*, beta-defensin 2, *Camp*, cathelicidin antimicrobial peptide, *Penk*, proenkephalin.  ^*∗*^*P*-value < 0.05.

Fold-change results indicated a significant increase in mRNA expression levels of *Kynu* (*P*=0.05, Figure 6(a)), *Defb2* (*P*=0.05, Figure 6(b)), *Camp* (*P*=0.034, Figure 6(c)), and *Penk* (*P*=0.05, Figure 6(d)) in the IMQ group versus the Vas group. The values in the IMQ group indicated fold changes of 2.70 ± 0.57, 4.56 ± 3.31, 3.29 ± 0.89, and 2.61 ± 0.47 relative to the Vas group, respectively. Also, the group received the M7 anti-IL-17A aptamer exhibited a significant decrease in *Kynu* (*P*=0.032, Figure 6(a)), *Defb2* (*P*=0.034, Figure 6(b)), *Camp* (*P*=0.021, Figure 6(c)), and *Penk* (*P*=0.034, Figure 6(d)) mRNA expression levels in comparison to the IMQ group. The results demonstrated 0.79 ± 0.34, 0.41 ± 0.20, 1.06 ± 0.20, and 0.63 ± 0.05-fold change relative to the Vas group, respectively. The correlation (Figure 6(e)) between *Kynu* and erythema (*r*; 0.807,*P*=0.008), *Penk* and erythema (*r*; 0.701, *P*=0.031) was significant. Also, *Camp* was significantly correlated with PASI (*r*; 0.752, *P*=0.011), erythema (*r*; 0.733, *P*=0.015), scaling (*r*; 0.756, *P*=0.010), and thickness (*r*; 0.713, *P*=0.018).

## 4. Discussion

We demonstrated that *Kynu*, *Defb2*, *Camp*, and *Penk* genes are upregulated by IMQ administration in a mouse model of psoriasis. Also, these gene expression levels were downregulated following mice treatment through inhibition of IL-17A with the anti-IL-17A aptamer. Additionally, these changes in *Kynu*, *Camp*, and *Penk* genes are related to the disease symptoms.

Psoriasis vulgaris is an inflammatory systemic disease with various manifestations [37]. The keratinocytes proliferation in psoriasis vulgaris is correlated with the alterations of inflammatory gene expression levels [38]. So far, studies have shown an association between psoriatic inflammation and changes in gene expression, such as *SERPINB4*, *DEFB4*, *S100A*, *CCL20*, *KRT16*, *KYNU*, *IL-6*, etc. [39]. Additionally, the gene expression of *IL-6*, *IL-1b*, and *IL-17* was investigated in the IMQ-induced psoriasis mice that were treated with anti-IL-17 aptamer [36]. Therefore, it appears that changes in the gene expression of *Kynu*, *Camp*, *Defb2*, and *Penk* are more appropriate candidates for the analysis of IMQ-induced psoriasis and anti-IL-17A aptamer treatment in this model. As a result, we investigated the changes in the gene expression levels of *Kynu*, *Defb2*, *Camp*, and *Penk* to determine which genes are more appropriate factors for the evaluation of psoriasis and the aptamer treatment efficacy. Among the animal models used for studying psoriasis, direct induction with 5% IMQ has been widely utilized in various basic research [40]. So, the IMQ was administered on the C57BL/6 mice skin in the IMQ group and in the aptamer-treated group following the M7 anti-IL-17A aptamer applied.

The evaluation of epidermal thickness and PASI scores on the 6th day of the experiment indicated a significant increase in the IMQ-treated mice and a decrease in the M7 anti-IL-17A aptamer-treated group. In this regard, it was observed that IMQ-induced psoriasis can be used as a mouse model for analyzing of psoriasis-like dermatitis pathogenesis and inflammatory cytokines [41]. Also, Shobeiri et al. [36] demonstrated that the M7 anti-IL-17A aptamer administration on the IMQ-induced psoriasis C57BL/6 mice dorsal skin ameliorated severity manifestations of skin psoriasis and reduced the *IL-17A*, *IL-1b*, and *S100a9* mRNA expression levels[36]. We noticed that our findings on epidermal thickness and PASI scores, in line with the aforementioned studies results, demonstrated that the psoriasis was induced following IMQ application. Additionally, the anti-IL-17A aptamer was able to ameliorated psoriasis symptoms.

CAMP and DEFB2 are two types of AMPs; they are synthesized in excessive quantities during psoriatic inflammation [42, 43]. In our research, *Camp* and *Defb2* expression levels in the IMQ-applied area showed around three and five fold increase, respectively. Additionally, in the M7 anti-IL-17A aptamer-treated group, these genes expressions were downregulated near to their levels in the Vas group. Wolfram et al. [44] measured the expression of the *Camp* in overexpressed KC-Tie2 mice's back skin by RT-PCR. The results indicated a significant upregulation in the *Camp* expression, they were reported 40-fold upregulation of *Camp* relative to the control group [44]. The local mouse CAMP levels of C57BL/6J mice dorsal skin exposed to IMQ, evaluated using immunoassay methods, revealed a significant 6.47-fold increase [45]. Kolbinger et al. [42] evaluated psoriatic patients before and after the administration of secukinumab and mentioned the expression of DEFB2 protein in the patient's lesional skin and sera increased, and after receiving secukinumab, along with the decrease in PASI score, the skin levels of DEFB2 were decreased. Also, the correlation was found between PASI disease activity and serum DEFB2 levels [42]. In line with Kolbinger study, our research showed decrement in *Defb2* expression levels after anti-IL-17 aptamer administration. Therefore, *Defb2* decrement can be considered as a marker in the inhibition of IL-17 effects. Nevertheless, we observed a significant reduction in *Camp* gene expression in the anti-IL-17A aptamer-treated group, which contrasts with the results of the Peric study. Peric et al. [46] demonstrated vitamin D analogs (calcipotriol) therapy improves human skin inflammation in psoriasis, but it strongly induces the expression of *CAMP* in the skin [46]. It seems that vitamin D analogs may ameliorate skin inflammation through pathways other than *CAMP* expression, and our treatment consequences may inhibit the effect of upregulated *Camp* in the development of the psoriatic manifestations.

It appears, another factor that demonstrates an upregulation in psoriatic inflammatory skin is KYNU. The KYNU enzyme is a part of the tryptophan metabolic pathway and is capable of eliciting inflammatory responses [47]. We observed that the *Kynu* mRNA expression in the IMQ-applied area demonstrated approximately a three fold increase, whereas results related to the M7 anti-IL-17A aptamer-treated group showed decreased this gene mRNA levels near to the Vas group. A meta-analysis-derived transcriptomic study has indicated that the *KYNU* gene increases an excess of 20-fold changes in psoriasis [48]. Moreover, KYNU is upregulated by TNF-*α* and IL-17 synergistically in the psoriasis [49]. Wang et al. [11] studied the BABL/c mice IMQ model, and their findings revealed that KYNU enzyme inhibition (by Carbidopa and Benserazide) reduced the inflammation of IMQ-applied mice dorsal skin [11]. Harden et al. [25] demonstrated the upregulation of the *KYNU* gene in the inflammation of human psoriasis and its reduction following successful therapy with etanercept or ustekinumab. Additionally, they revealed a positive correlation between *KYNU* expression and PASI score. According to publicly available gene array data, indoleamine-2,3-dioxygenase (*IDO*) is overexpressed compared to the *KYNU* in cancers such as ovarian, lung, and cervical, while in inflammatory diseases, *KYNU* is overexpressed. Also, the *KYNU/IDO-TDO* ratio demonstrated a significant increase in inflammatory diseases [25]. IDO promotes an anti-inflammatory environment in the skin by T-cell suppression and regulatory T-cell induction [50], which in turn reduces *KYNU* expression levels [25]. Therefore, this appears that inhibiting the IL-17 in an aptamer-treated group helps the modulation of an inflammatory milieu in the skin, thereby may affecting tryptophan metabolism through the IDO.

The mRNA expression of the *PENK* increases in inflamed skin [23]. PENK is a precursor of ENKs, which are endogenous analgesics [51]. The PENK is a neurotransmitter involved in pruritus and nociception. It is also known as an AMP [20, 21]. Because ENKs are mediators of inflammation and induce pruritus [52], it is expected this mediator increased in psoriasis condition. Our results of *Penk* expression levels evaluating in the IMQ-applied skin indicated an approximately three fold increment. Also, it decreased in the M7 anti-IL-17A aptamer-treated group to near the Vas group level. Oishi et al. [53] performed a comprehensive analysis on the pruritus in IMQ-induced psoriasis BALB/c mice. The results revealed the upregulation of preproenkephalin (*PPE*) mRNA expression to an excess of 295-fold change in the IMQ-applied mice in comparison to the mice without IMQ [53]. Loite et al. [54] indicated that the levels of the *PENK* mRNA expression in the human psoriatic lesional skin versus nonlesional skin were 1.6 times higher[54]. Furthermore, another research accomplished by Slominski et al. [23] showed that the *PENK* was significantly expressed in human skin keratinocytes. Also, Met/Leu-ENK expression increased in psoriasis. Nissen et al. [22] applied calcipotriol and mometasone furoate on the psoriatic patient's skin and then measured ENK in the skin by using the radioimmunoassay method. They found that the levels of ENK were increased in psoriatic lesions and decreased after clinical improvement caused by treatment [22]. Consequently, our findings are in line with the aforementioned studies showing that the *Penk* mRNA expression levels are an appropriate factor in assessing psoriasis.

Our findings indicated that in addition to increased expression levels of *Camp*, *Defb2*, *Kynu*, and *Penk* in the IMQ group, PASI and epidermal thickness scores also increased. Moreover, these measures decreased following treatment with the aptamer. Harden et al. [25] demonstrated that the upregulation of *KYNU* mRNA in psoriasis-affected skin is associated with an increase in PASI score [25]. H&E staining analysis showed an increase in epidermal thickness of BALB/c mice treated with IMQ on the 4th day of the administration procedure, along with *Kynu* mRNA expression levels increment [11]. Additionally, Salamah et al. [45], in agreement with our findings, indicated that CAMP skin levels and PASI scores increased in the C57/BL6J mice undertreated with IMQ.

IMQ is an TLR7/8 agonist, which entry to the circulation after topical administration and leads to a systemic inflammation through the increased of inflammatory cytokines mRNA expression, especially *TNF-α* in the mice lungs and liver [55]. Jabeen et al.[56] demonstrated an association between elevated TNF-*α* serum levels and spleen length. Furthermore, the enlargement and germinal center activity increment of the lymph node indicated the IMQ systemic effects. Spleen and lymph nodes are secondary lymphoid organs that can be associated with the psoriatic inflammation [56]. It appears that IMQ-induced systemic psoriatic inflammation increases the spleen mass, splenocyte count, and size of the spleen and lymph node [56, 57, 58], which reverses after treatment [59]. Our findings showed that spleen and lymph node indexes increased in the IMQ group, while the systemic effects of IMQ were not a notable improvement in comparison to the local effect of anti-IL-17 aptamer in the M7 aptamer group. In our study, the skin local inflammation induced by IMQ leads to skin *Camp*, *Defb2*, *Kynu*, and *Penk* mRNA expression increment along with effects on the spleen and lymph node. Also, anti-IL-17 local administration reduced the skin mRNA expression levels significantly, but the spleen and lymph node indexes slightly decreased. It seems acceptable because the targeting inhibition of immune pathways locally should not affect the systemic inflammation.

## 5. Conclusion

The results of our investigation in *Camp*, *Defb2*, *Kynu*, and *Penk* gene expression levels proved that changes in these genes occur in IMQ-induced psoriasis. Also, treatment with the anti-IL-17 aptamer showed a decrease in the expression of our studied genes. Interestingly, alterations in *Camp*, *Kynu*, and *Penk* are closely positively correlated with clinical symptoms, and *Camp* showing a stronger correlation. In conclusion, *Camp*, *Kynu*, and *Penk* can be used as markers to assess the induction of psoriasis vulgaris model and treatment effectiveness.

## Figures and Tables

**Table 1:** The primers were designed for the evaluation of *Kynu*, *Camp*, *Defb2*, and *Penk* mRNA expression levels.

Gene	Accession number	F primer (5ʹ−3ʹ)	R primer (5ʹ−3ʹ)	Product length (bp)
Mouse *Kynu*	NM_027552.3	CACGGACTTGATGTTGAGAA	TTTGTTATGGCAGGAATGTTG	179
Mouse *Defb2*	NM_010030.2	CTGATATGCTGCCTCCTT	AGTTCTGCTTCGTATCCAA	77
Mouse *Camp*	NM_009921.2	TGTATGTGGCAAGGCAGAG	GCACCAGGCTCGTTACAG	138
Mouse *Penk*	NM_001002927.3	AGGCGACATCAATTTCCTG	AGATCCTTGCAGGTCTCC	84

## Data Availability

The data that support the findings of this study are available from the corresponding author upon reasonable request.
